# Children’s Physical Activity Behavior during School Recess: A Pilot Study Using GPS, Accelerometer, Participant Observation, and Go-Along Interview

**DOI:** 10.1371/journal.pone.0148786

**Published:** 2016-02-09

**Authors:** Charlotte Skau Pawlowski, Henriette Bondo Andersen, Jens Troelsen, Jasper Schipperijn

**Affiliations:** 1 Research Unit for Active Living, Department of Sports Science and Clinical Biomechanics, University of Southern Denmark, Campusvej 55, 5230 Odense M, Denmark; 2 Centre for Intervention Research in Health Promotion and Disease Prevention, National Institute of Public Health, University of Southern Denmark, Øster Farimagsgade 5a, 1353 Copenhagen K, Denmark; Universidad Europea de Madrid, SPAIN

## Abstract

Schoolyards are recognized as important settings for physical activity interventions during recess. However, varying results have been reported. This pilot study was conducted to gain in-depth knowledge of children’s physical activity behavior during recess using a mixed-methods approach combining quantitative GPS and accelerometer measurements with qualitative go-along group interviews and participant observations. Data were collected during three weekdays in a public school in Denmark. Eighty-one children (47 girls) wore an accelerometer (ActiGraph GT3X) and GPS (QStarz BT-Q1000xt), sixteen children participated in go-along group interviews, and recess behavior was observed using an ethnographical participant observation approach. All data were analyzed separated systematically answering the Five W Questions. Children were categorized into Low, Middle and High physical activity groups and these groups were predominantly staying in three different locations during recess: school building, schoolyard and field, respectively. Mostly girls were in the building remaining in there because of a perceived lack of attractive outdoor play facilities. The children in the schoolyard were predominantly girls who preferred the schoolyard over the field to avoid the competitive soccer games on the field whereas boys dominated the field playing soccer. Using a mixed-methods approach to investigate children’s physical activity behavior during recess helped gain in-depth knowledge that can aid development of future interventions in the school environment.

## Introduction

The physical, mental, and social health benefits of physical activity (PA) in children are well documented [1, 2]. Despite the benefits of PA, a significant number of children in Denmark and other Western countries do not reach recommended levels of PA [3, 4].

As recess PA has been reported to contribute with up to 40% of children’s recommended daily PA [5], the physical environment of the school has long been recognized as an effective setting for PA initiatives, particularly schoolyards during recess [6, 7]. However, PA behavior during recess can vary widely depending on schoolyard space [8–11], facilities [12, 13], gender [14, 15] and social grouping [16, 17]. Moreover, studies in school-based PA interventions have reported varying results concluding that an in-depth exploration of children’s PA behavior during recess is needed [6, 18–22].

To grasp the complexity in PA behavior in schoolyards the current study builds on a dual-process view on the environment–behavior relationship conceptualized in a model by Kremers et al. and modified by Troelsen positing that PA behavior is influenced of conscious and unconscious processes related to the environment [23, 24].

PA levels and behavior during recess have been measured primarily using quantitative measurements such as accelerometers or self-reported data in previous studies [6, 25–28]. When assessing location and intensity of play behavior in schoolyard environments the majority of studies have used the System for Observing Play and Leisure Activity in Youth (SOPLAY) [29–33]. Some studies are based on a combination of quantitative methods (heart rate or accelerometer combined with GPS) to objectively describe children’s PA behavior and location during recess [15, 34–36]. Two other quantitative techniques have been used to examine children’s recess behavior. The System for Observing Children's Activity and Relationships during Play (SOCARP) providing information on children’s behavior and social interactions [9] and a write and draw technique to examine what children like and dislike about recess [37]. To get an understanding of children’s behavior, social interaction and perceived PA during recess other studies have used qualitative-phenomenological approaches such as different interview techniques [8, 10, 16, 38] and ethnographical observation approaches [16, 39–41]. To our knowledge only two studies on PA behavior during recess have used a mixed methods approach combining SOPLAY with focus group interviews [42] and systematic observations with questionnaires [43], respectively.

Each research method has its advantages and limitations in exploring children’s PA behavior during recess. However, none of the previous studies exploring children’s recess PA behavior have systematically combined objective measurements such as GPS and accelerometer with qualitative methods. A mixed methods approach has the potential to provide an in-depth knowledge of children’s PA behavior [44]. Providing a more complete picture of children’s PA behavior during recess can further qualify e.g., intervention studies and natural experiments as it is when behaviors and the environment are understood that effective interventions can be designed [23, 45].

The aim of this study was to gain in-depth knowledge of children’s PA behavior during recess by pilot testing a mixed-methods approach combining the quantitative measurements GPS and accelerometer with qualitative go-along group interviews and participant observations.

## Method

### Setting

This study was carried out at a public school in a rural lower middle class area in the western part of Denmark. 381 students were enrolled at the school divided into junior (grade 0–3), middle (grade 4–6) and senior (grade 7–9) tiers. Almost all students were ethnic Danes (99%). Our target group consisted of the middle tier students (grade 4–6; 10–13 years-old) in order to get a better understanding of PA behavior among an age group that is known to significantly decrease their PA [46].

The school grounds covered 13,311 square meter (35 square meters per child) and were separated in a paved schoolyard with play markings, a large grass area with soccer fields and a well-equipped playground for junior students only. During the school day there were three breaks all included in our study; morning tea and lunch break lasting 30 minutes each, and a 10 minutes afternoon break. All breaks were characterized by free play supervised by teachers. The junior students must stay outdoors during recess but the school had no outdoor recess policy for middle and senior tier students. Classrooms, corridors, a library and a canteen were the indoor areas allowed to be used by middle and senior tier students during recess.

The school was recruited to the current study as part of the baseline study of a schoolyard intervention study: The Activating Schoolyards Study [47]. This study aims to get knowledge about how to improve children’s opportunities to become physically active in the schoolyard during recess, in particular the least physically active schoolchildren. The current study was conducted prior to the implementation of the schoolyard intervention. The school is similar to many other Danish schools in terms of the type of school buildings, size, recess organization, characteristics of school grounds, and number of students enrolled [8].

### Recruitment

Eighty-five (48 girls) out of 115 children attending the middle tier agreed to participate in the study by wearing accelerometer and GPS. Three go-along group interviews (one for each middle tier grade level) were conducted. Participants were purposely sampled with help from a designated middle tier teacher who was able to recruit children from the middle tier classes with diverse characteristics to ensure variation in gender, social backgrounds and PA level to allow for contrasting opinions. This approach was employed to ensure both homogeneity and heterogeneity within the groups [48, 49]. In total 16 children (eight girls) participated in the go-along group interviews. The group-size ranged from four to six participants (six participants in the interview with grade 4 and 5 children and four participants in the interview with grade 6 children). Group interviews with four to six participants are recommendable if the study is to gain in-depth insight of people’s experiences. Also, smaller groups are preferable when the participants have a great deal to share about the topic or have had intense or lengthy experiences with the topic of discussion [48, 49].

#### Ethical approval

All parents of the participating children provided a written informed consent on behalf of the children, and all children could withdraw from the study at any time. Parents of the 16 children participating in go-along group interviews provided and additional written informed consent for the interview. Data were collected in accordance with the Helsinki declaration and this type of consent procedure has been found to be ethically appropriate in low-risk research in children at the age group enrolled in our study [50]. According to the Danish National Committee on Health Research Ethics formal ethical approval was not required as the project was not a biomedical research project. The study and its data-management procedures have been approved by the Danish Data Protection Agency (2013-41-1900 and 2014-41-2801).

### Data collection and measurements

All data were collected during three schooldays in June 2014. The study used four different data collection methods and measures with specific aims in relation to explore the children’s PA behavior, as described in more detail below.

#### Accelerometer and GPS

Objective PA data were recorded as an activity-count every 15 seconds using the ActiGraph accelerometer model GT3X to explore differences in PA intensity between grade, gender and recess periods [51, 52]. We did not use the low frequency extension (LFE) option during data collection.

The children’s locations during recess were measured every 15 seconds using QStarz BT-Q1000xt GPS trackers [53]. The schoolyard was mapped in detail using the Geographic Information System (GIS) software ArcGIS 10.3 and the total outdoor area at the school was calculated.

The children were asked to wear the accelerometer and GPS in an adjustable elastic belt on their waist during the data collection period. Verbal and written instructions on how to wear the equipment were given to the children by the research team. The equipment was not worn overnight and during water-based activities. To increase compliance the children received short reminder text-messages on their mobile phones twice a day.

#### Participant observation

Participant observation, an ethnographical observation approach [54], was used to gain insight in children’s PA behavior during recess by exploring types of activities and interactions at different locations and recess periods. The observations were conducted during recess on three weekdays where the children wore accelerometer and GPS.

The participant observations were focused on the middle tier students (grade 4–6) wearing accelerometer and GPS by following these children around in different outdoor and indoor areas. The observations were driven by an open approach to the explored field [55]. This lead to observations of both specific activities and specific groups of children. The observer either participated actively in the children’s activities, or passively observed the children from a distance. The researcher’s position was adapted to fit the situation [54]. Observations were documented with field notes and photos [56].

#### Go-along group interview

We conducted group interviews with the selected children to explore the children’s subjective perceptions and attitudes to their PA behavior during recess [57, 58]. To facilitate the conversation and evoke memories the interviews were carried out walking around in the schoolyard inspired by a go-along interview approach [59, 60].

The tree go-along group interviews were conducted during lessons. The go-along group interviews lasted for approximately 60 minutes. Prompts during the walk included for example: ‘What do you do during recess?’ ‘With whom are you doing it’? ‘Where are you doing it’? ‘When are you doing it’? ‘Why are you doing it?’ The go-along group interviews were filmed using an iPad mini2® to record both verbal and nonverbal interaction of the children.

### Analysis

The quantitative and qualitative data were analyzed separately. In all analyses we systematically used the Five W Questions as an underlying analytical tool to reveal a more complete story on PA behavior during school recess (Who did that? What happened? When did it take place? Where did it take place? And Why did that happen?) [61]. ‘Where’ the children were during recess was used to drive the first step in both data analysis.

#### Quantitative analysis

At the end of the data collection period the accelerometer and GPA data were downloaded using ActiLife v.6.11.4 and GPS data logger software BT747 (www.bt747.org), respectively. All accelerometer and GPS files were processed using the Personal Activity and Location Measurement System (PALMS, https://ucsd-palms-project.wikispaces.com) to match the two types of data based on their timestamp and calculate wear time and PA. The Evenson cutpoints, which have been recommended to estimate PA intensities among children, were used to classify moderate-to-vigorous physical activity (MVPA) [62, 63]. Continuous periods of 60 min of zero values were classified as accelerometer non-wear time, and were removed from the data [64]. The combined data were then downloaded into a PostgreSQL database and combined with data from class timetables and schoolyard GIS data. In the database, school time was selected for each participant based on the class timetables.

Eighty-one children (47 girls) with combined accelerometer and GPS data during all recess periods on the three days of data collection were included in the analysis. For each recess period we ran a ‘hot-spot analysis’ in ArcGIS 10.2 in order to find locations that were important for activity. The ‘hot-spot analysis’ tool was used to calculate the Getis-Ord Gi* statistic [65] for the activity count values of each GPS-point. This tool works by looking at each feature within the context of neighboring features. A point with a high activity count value is interesting but may not be a statistically significant hot spot. To be a statistically significant hot-spot, a point will have a high activity count value and be surrounded by other points with high activity count values.

Based on mean MVPA per child during recess three activity groups were created. The Low PA group represents children within the lowest activity quartile, the Middle PA group consisted of children in the middle two quartiles and children in the highest quartile were grouped in the High PA group.

The statistical analyses were performed using STATA SE13. To describe time and PA level per area type (defined by the hot-spot analysis) descriptive statistics were calculated using median and interquartile ranges (IQR) for each activity group, as these variables were not normally distributed.

#### Qualitative analysis

To ensure consistency, the first author transcribed all observation field notes and interviews. Then field notes, photos, and interview transcripts were coded to identify different locations for children’s recess PA. Afterwards we analyzed the PA behavior in each of the locations by answering Who, What, When and Why questions to create a deeper understanding of the data. Using a thematic analysis, building on a coding and re-coding process based on similarities and variations in the material, a set of analytical categories emerged [66]. In the presentation of the results children were anonymized by using pseudonyms.

## Results

In total 81 participants were included in the analyses and 22 (15 girls) belonged to the lowest activity quartile (Low), 38 (30 girls) to the middle two quartiles (Middle) and 21 (2 girls) to the highest quartile (High) based on minutes spent in MVPA during recess. The median time spent in MVPA during recess was 2.8, 8.3 and 19.9 minutes for the Low, Middle and High PA group, respectively. During the whole school day the median time spent in MVPA was 54.0 minutes, 75.6 minutes and 87.0 minutes respectively.

**Fig 1:**
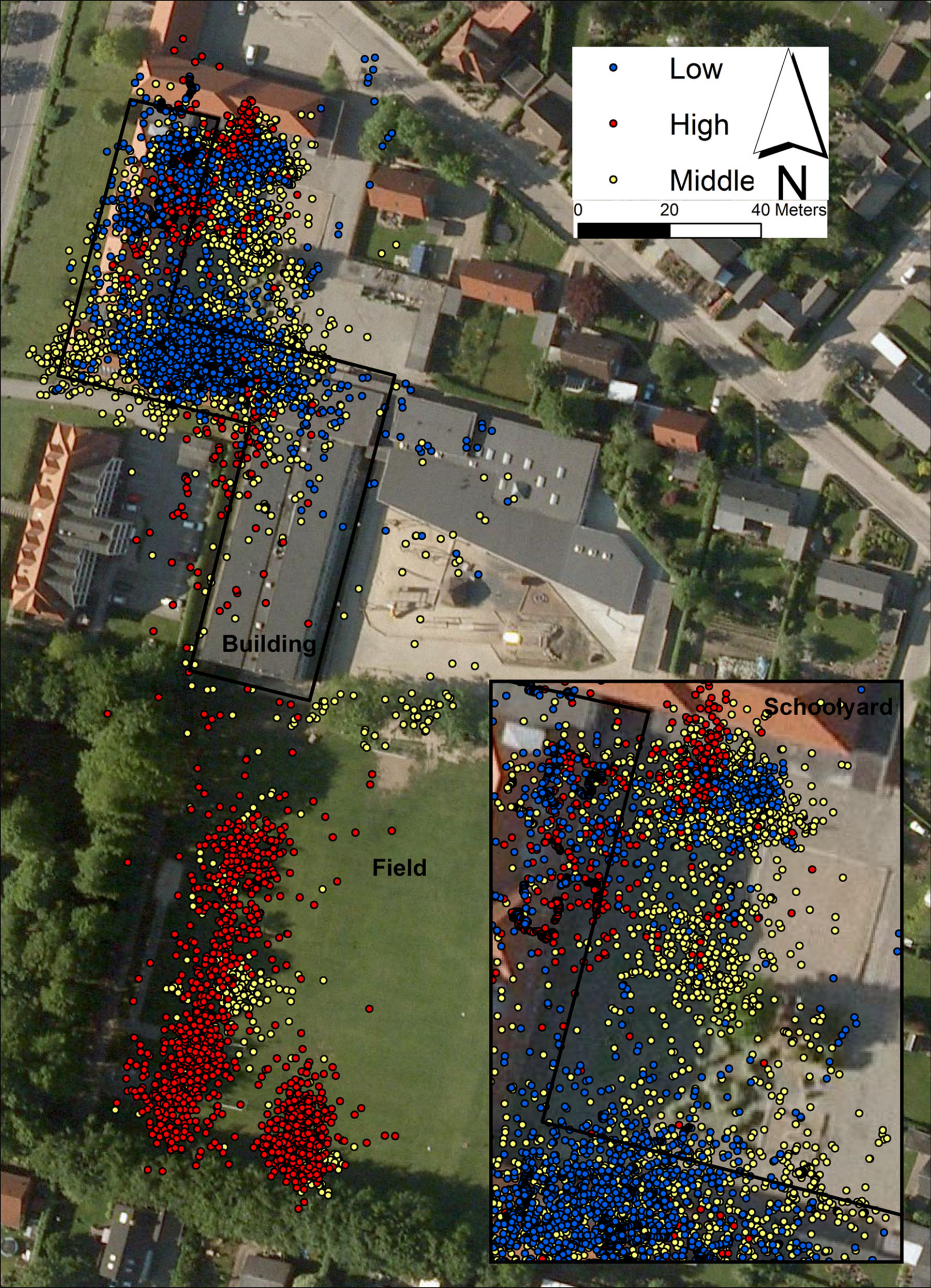
An example of where the Low, Middle and High PA groups were during recess. Contains data from the Danish Geodata Agency, Ortofoto, WMS.

Based on both analyses three main locations could be distinguished: building (i.e., the entire indoor school area), schoolyard and field. The building was a cold-spot (low activity spot), the schoolyard had both cold-spots, e.g., a skate-board ramp and balancing bars used as hang-out area, as well as hot-spots (high activity spots), e.g., a foursquare area, and the field was a hot-spot (Fig 1).

All children spent at least some time in the building, 79 out of 81 children visited the schoolyard during recess and 51 children visited the field. A more specific analysis of the three locations will be presented below.

### Building

All children spend some of their recess time in the building, but the median time spent varied between the three groups. Children in the Low PA group spent most time in the building with a median of 44.1 minutes. The Middle PA group spent a median of 24.2 minutes there and children belonging to the High PA group spent a median of 12.5 minutes inside (Table 1).

**Table 1:** Characteristics of time spent, activity level and behavior in the building during recess.

Building		Quantitative		Qualitative
Who	Low n = 22 (15 girls)	Middle n = 38 (30 girls)	High n = 21(2 girls)	Few boys and a predominance of girls.
What MVPA lightSed	Median (IQR) 1.2 (0–2.8) 9.3 (0.8–22.7)27.0 (3.3–41.9)	Median (IQR) 1.9 (0.3–4.3) 8.7 (3.4–19.9) 14.1 (1.4–27.4)	Median (IQR) 2.3 (0.8–4.1)4.2 (0.9–8.5) 6.3 (0.8–11.1)	Sedentary activities such as playing computer games, mobile phone or cards, reading books, painting and hanging around talking.
When Time	Median (IQR) 44.1 (4.5–51.9)	Median (IQR) 24.2 (7.3–41.5)	Median (IQR) 12.5 (5–21.9)	Was used during all recess periods.
Where	Most of the indoor staying children were in the area for middle tier students including classrooms, corridors and a canteen			Classrooms, corridors, canteen and library.
Why				Most preferred to stay outdoors but stayed indoors because they experienced a lack of outdoor facilities appealing to them.

Median, IQR and Time all in minutes Low, children in the lowest activity quartile; Middle, children in the middle two activity quartiles; High, children in the highest activity quartile. Activity quartiles are generated based on mean physical activity during recess. IQR, Inter Quartile Range; MVPA, Moderate to Vigorous Physical Activity; Light, Light activity; Sed, Sedentary activity

According to the observations we mostly found the children staying in four different zones in the school building: classrooms, corridors, canteen and library. Based on observation and the ‘hot-spot’ analysis these areas seemed to appeal to sedentary activity. The classrooms were small and packed with desks and chairs and the corridors were narrow but had some couches and small niches with tables and chairs. In the library and canteen the children were only allowed to sit down quietly during recess.

The median time spent sedentary in the building was 27.0, 14.1 and 6.3 minutes for the Low, Middle and High PA group, respectively. In contrast, time spent in MVPA was low for all three groups with 1.2, 1.9 and 2.3 minutes respectively.

When the bell rang we observed that a few boys and girls remained seated quietly in their classroom and became absorbed in their own sedentary activity during the whole recess. The boys typically started playing computer games whereas the girls started reading books or began painting. However, most of the children indoors were girls socializing with their classmates in smaller groups by talking, playing cards or walking around.

Based on the interviews, most of the children staying indoors expressed that they did not stay indoors because they were attracted to the indoor area as a place, but because they felt a lack of motivating outdoor play facilities. Many girls often felt bored and hung around indoors because they did not know what to do during recess, as exemplified below:

Moderator: Do you arrange things to do during recess?

Maya: No, I just sit indoors with my girlfriends. We just sit indoors talking because there is not so much to do outdoors.

Andy: You fool around, throw food and yell.

Moderator: Are you happy doing this or do you prefer doing something else?

Maya: Well, there is not really anything to do during recess and if we go outdoors you can only play soccer. But sometimes we take a quick walk in the schoolyard and then we walk back to our classroom again to sit down talking.

Many children expressed that if there were more different play facilities in the schoolyard they would prefer to do activities outdoors during recess.

### Schoolyard

The schoolyard was an asphalt-paved square enclosed by school buildings. In the northern part, the square consisted of a zone with two marked foursquare pitches. In the middle of the square a basketball zone, a small multi court, and a picnic table were placed. The southern part of the schoolyard consisted of a small area with gravel, big stones and balancing bars, two small marked soccer pitches, and a ramp for skateboarding.

Almost all children spent some time in the schoolyard with a median of 7.0 minutes for the Low PA group, 16.4 minutes for the Middle PA group, and 3.1 minutes for the High PA group. The median time spent in MVPA was 0.8, 3.6 and 0.5 minutes for the three groups, respectively. The children, predominantly girls, in the Middle PA group that spent most time in the schoolyard spent a median of 7.8 minutes in light activity and 4.1 minutes sedentary. Many of these girls were only in the schoolyard for a short period of time, walking in and out of the building. They explained that they primarily used the schoolyard because it was the easiest outdoor area to reach from their respective classrooms. During the short afternoon break also many boys were in the schoolyard because the break was too short (10 minutes) to get to the field and start-up a soccer game (Table 2).

**Table 2:** Characteristics of time spent, activity level and behavior in the Schoolyard during recess.

Schoolyard		Quantitative		Qualitative
Who	Low n = 21 (14 girls)	Middle n = 35(28 girls)	High n = 21 (2 girls)	Particularly girls from all middle grade classes and few boys.
What MVPA lightSed	Median (IQR) 0.8 (0–3)2.7 (0–16.3)3.5 (0–11.8)	Median (IQR) 3.6 (0–9.3)7.8 (0.1–26.6)4.1 (0–12.2)	Median (IQR) 0.5 (0–22.3)1.3 (0–29.9)0.9 (0–6.9)	Foursquare, shooting hoops, a ‘playing horse’ play, walking around and hanging out.
When Time	Median (IQR)7.0 (0.1–29.6)	Median (IQR) 16.4 (0.3–44.4)	Median (IQR) 13.1 (0.2–48)	Is used during all breaks but most during the short afternoon break because the break is too short to get to the field and start up a soccer game.
Where	The Low PA group is typically in the northern part or close to the building entrances southwards. The Middle PA group uses the whole area but mostly the middle and northern part. The High PA group typically uses the northern part.			Foursquare and shooting hoops were taking place in the northern and middle part of the schoolyard, respectively. The ‘playing horse’ play was taking place on a soccer pitch in the southern area. Hanging out was primarily done on a skateboard ramp and at balancing bars in the southern part. The walking activity was done all over the schoolyard.
Why				It was the easiest outdoor area to reach from the classrooms and the games played in the schoolyard were experienced less serious and more gender-inclusive than the soccer games on the field.

Median, IQR and Time all in minutes. Low, children in the lowest activity quartile; Middle, children in the middle two activity quartiles; High, children in the highest activity quartile. Activity quartiles are generated based on mean physical activity during recess. IQR, Inter Quartile Range; MVPA, Moderate to Vigorous Physical Activity; Light, Light activity; Sed, Sedentary activity

‘Waiting’ was a frequent activity in the schoolyard. The basketball hoops were used by a group of girls who were shooting hoops one at a time while the others were waiting their turn at the picnic table. Furthermore, foursquare games were a popular activity in the schoolyard, but since only four children could actively participate at one time, many children were waiting in line for their turn to play. From the interviews it became clear that many of the foursquare-playing children chose this over playing soccer on the field because they found soccer too serious, too competitive and often too conflict-ridden. They wanted to play for fun and preferred the gender and age mixed play and therefore chose to play foursquare. In line with the children’s statements we observed more laughing and flirting in the foursquare zone than foursquare play.

The small multi court was empty during most of the breaks. The children explained that they needed a special type of ball and that all these balls had disappeared. Rather unexpectedly, we observed four girls pretending to be horse riding and imitating horse dressage using one of the soccer pitches:

Cathirne: I mostly play horse with my girlfriend.

Moderator: Where are you playing that?

Cathrine: In the schoolyard.

Moderator: How do you play horse?

Cathrine: We jump or do dressage or something like that.

Moderator: Are there reasons why you do it in the schoolyard?

Cathrine: Yes, there are marked lines for soccer, which we can use for the horse dressage.

We also observed a number of smaller groups of girls hanging around in the schoolyard talking. Some were sedentary sitting on the skateboard ramp or at the picnic table and some were standing on the balancing bars. Girls were also observed just walking around the schoolyard often arm in arm. Sometimes they stopped for a while talking with some of the girls hanging out before they continued walking in the schoolyard or went indoors.

### Field

The field area was verbalized as the “happening” recess location. 20 children (2 girls) belonging to the High PA group were on the field, with a median stay of 35.4 minutes. Their median time spent in MVPA was 14.0 minutes, while a median of 16.0 minutes were spent in light activity. For the Middle PA group, 23 children (18 girls) visited the field, with a median stay of 10.9 minutes, 1.3 minutes in MVPA and 5.2 minutes in light activity. For the 8 children (6 girls) categorized in the Low PA group that visited the field, their median stay was less than a minute (Table 3).

**Table 3:** Characteristics of time spent, activity level and behavior on the field during recess.

Field		Quantitative		Qualitative
Who	Low n = 8 (6 girls)	Middle n = 23(18 girls)	High n = 20 (2 girls)	Both boys and girls but a preponderance of boys. They preferred to play class-divided.
What MVPA lightSed	Median (IQR) 0.4 (0–2.7)0.3 (0–2.8)0 (0–1)	Median (IQR) 1.3 (0.1–7.1)5.2 (0.3–25.6)1.3 (0.1–10.3)	Median (IQR) 14 (7.5–23.8)16 (3.5–28.3)2.5 (0.1–8.5)	Soccer was the main activity. Hanging out either sedentary or by walking around was a secondary activity.
When Time	Median (IQR)0.8 (0.1–5.7)	Median (IQR) 10.9 (0.5–35.6)	Median (IQR) 35.4 (12.3–47)	Was used during the two main recess periods.
Where	The Middle and High PA group mostly use the southwestern part of the field. The Low PA group is predominantly in the northern part of the field close to the building.			Soccer was played at up to four different soccer fields. The hanging out activity occurred at the sidelines.
Why				Experienced as the most “popular” place in the school ground and one of the only locations with facilities to do recess activities. Moreover, the grassy grounds made it possible to play “real” soccer and tackle without hurting oneself.

Median, IQR and Time all in minutes. Low, children in the lowest activity quartile; Middle, children in the middle two activity quartiles; High, children in the highest activity quartile. Activity quartiles are generated based on mean physical activity during recess. IQR, Inter Quartile Range; MVPA, Moderate to Vigorous Physical Activity; Light, Light activity; Sed, Sedentary activity

The field was set-up for soccer with four marked soccer fields and soccer goals in different sizes. The grassy grounds were attractive for playing soccer because it was possible to play “real” soccer and tackle without hurting oneself. The children experienced that soccer on the field was one of the few recess activities on the school grounds. Three to four soccer games were played at the same time, primarily during the two main recess periods. Most children preferred to play soccer solely with their classmates. But because of a clear quality hierarchy of the soccer fields they sometimes had to play soccer with children from other classes and grades to play at the best fields placed in the southwestern part of the field. In addition, a grade hierarchy was found in the fight for getting the most attractive soccer fields as shown below:

Alex: If there are some younger students then you can just get rid of them. You can take their goals and annoy them until they leave.

Moderator: Is that what you are doing?

Tom: Yes

Katia: You shouldn’t say that, dimwit

Moderator: Is that what the older students do to you as well?

Alex: Yes

The fight for getting the most attractive fields often caused conflicts which the school managed by keeping the involved children in quarantine from the field a couple of days.

Boys as well as a few skilled girls primarily played the soccer games. The field was experienced as a boys’ domain, even though also many girls visited the field. Girls stated that it was not motivating to play soccer with the boys because the boys did not pass the ball to them unless they were skilled. Instead most girls observed on the field kept to the sidelines hanging out in smaller groups, possibly because of the status connected with being at the most “happening” place. Some were sitting down watching the game or talking while others were walking around talking and now and then they did some dancing or gymnastic moves.

## Discussion

Various studies have emphasized the need for comprehensive explorations of children’s PA behavior during recess in order to inform future schoolyard interventions [6, 18–22]. This study contributes to the current literature with an in-depth investigation of children’s PA behavior during recess combining the quantitative measurements GPS and accelerometer with qualitative go-along group interviews and participant observations.

In line with Kremers et al. and Troelsen claiming that specific behavioral determinants of energy balanced-related behaviors including PA will differ for different groups [23, 24] our results revealed that children displayed different PA behavior during recess. Also, the children’s PA level was associated with their location in the school environment showing a relationship between environment and behavior [24].

Two-thirds of the children belonging to the Low PA group were girls involved in sedentary socializing activities in the classroom. As main reason for their behavior the children expressed a lack of attractive outdoor activity possibilities. Thirty out of the 38 children in the Middle PA group were girls, and many of them were engaged in walk-and-talk behavior, both in the schoolyard and on the field. This group was furthermore involved in foursquare games, shooting hoops, and pretending to be horse riding. Arguments used by the children as to why they were involved in these activities were that they preferred gender and age mixed play, and wanted to avoid the many conflicts associated with soccer. Boys dominated the High PA group and spent most of their time on the field playing soccer. Approximately 50% of their recess time was spent in MVPA. This group took soccer very seriously and a high level of skills was needed to participate in the game.

### What to do when planning interventions?

The study gave us an understanding of three groups of children with varied PA behavior and use of different locations during recess. Even though our study was conducted as a pilot study, current data combined with previous findings gave us more insight into how to tailor future interventions to increase recess PA across different groups of children. According to the modified model by Kremers et al. altering environmental determinants in the school setting can influence the children’s PA behavior both indirectly and directly [24]. For that reason we will focus on interventions altering the environment in the below suggestions.

When planning to alter PA behavior among the Low PA group, mostly staying indoors, it is important to recognize that they explained that they stayed indoors during recess because of an experienced lack of outdoor play facilities. This perceived lack of outdoor facilities is in line with a number of studies [8, 10, 16, 38]. A review also found a positive association between recess PA and overall facility provision as well as the provision of unfixed equipment [14]. According to the model by Kremers et al. involving the children in making decisions on what kind of facilities should be implemented would be an important factor in changing their PA behavior [23]. Since we found higher PA levels outdoors, implementation of a ‘be outdoors during recess’ policy could possibly be a strategy to increase the PA level among the Low PA group by directly influencing their automatic, unconscious behavior [23]. Other studies also found that outdoor school environments facilitated play and were associated with increased levels of recess PA [4, 34, 67].

The Middle PA group generated mostly light PA during recess and was predominantly found in the schoolyard. In the schoolyard we found waiting time as a restricting factor for PA due to the limited number of facilities in relation to the number of children wanting to use them (e.g., foursquare pitches and basket hoops). Other studies have found that the number of school-ground play facilities is associated with the daily amount of PA [13, 68, 69]. We also found that the small multi-court was unused due to a lack of suitable balls. Access to more and different kinds of balls would possibly change PA behavior and generate more PA in the schoolyard. Zask et al. reported that the ratio of balls to children was related to vigorous physical activity (VPA) during recess [70]. Moreover, we found children using facilities differently than expected (e.g., pretending to be horse riding on a soccer pitch), which might call for more variation in facilities. Children asking for more variation in facilities was also found in another study [8].

The third group of children, mostly boys, generated a relatively high amount of MVPA during recess at the soccer field. A previous study also found that time spend in MVPA was highest at the field compared to the playground [4]. This group did not seem in need of any PA stimulating intervention. However, the boys’ highly competitive behaviour and the many conflicts occurring at the soccer field could have a negative impact on the children’s PA. In a previous study, conflicts were perceived as time consuming and a barrier to recess PA, especially among competitive sports-minded boys [8]. The lack of teacher presence in outdoor areas seems to be related to conflicts, hence increased teacher supervision might lead to faster conflict resolution and provide increased PA, particularly among boys [42, 71]. Similar to a previous study we found that girls wanted to play soccer but felt excluded [16]. Teacher organized recess activities such as soccer could be an initiative to indirectly stimulate involvement of girls in boys’ activities, and vice versa, by influencing, what Kremers et al. called, the children’s mediators of behavior-specific cognitions [23]. In line with this, a study found that when trained teachers initiated recess activities, this was associated with increased PA [72].

### Was mixing all these methods really necessary?

The current study had both strengths and limitations. Mixing four methods is a complex and time-consuming process requiring a high level of resources. However, we found that the mixed methods approach strengthened the study by facilitating a much richer form of data and created a greater credibility of results by offering complementary insights and understandings that neither the quantitative nor qualitative methods alone had the potential to achieve.

The quantitative methods could not identify what kind of activities the children were doing and why they were doing them. As an example the children’s PA level at a soccer area in the schoolyard was identified by using accelerometers and GPS but by these methods we could not conclude what they were doing. Unexpectedly, we observed the soccer area being used by girls pretending to be horse riding. Systematic observations like SOPLAY and SOCARP can record both PA levels in open environments and find what the children are doing. But these methods cannot be used to analyze behavior for specific groups due to the lack of background information on the children observed (e.g., PA level, age) [73]. Furthermore, all these methods have limitations in creating an understanding of the factors affecting children’s PA behavior. To really understand the children’s PA behavior it is crucial also to use qualitative methods [57]. Aside from determining what the children are doing, participant observations can provide insight into children’s social relations during activities [54]. Additionally, interviews with the children can reveal the children’s perceptions and give explanations of behavior, as for example in this study where a perceived lack of outdoor facilities was given as reason for staying indoors. Particularly, a child participatory method such as the go-along group interview is valuable to capture children’s perceptions of PA [57, 74, 75]. However, also the qualitative methods cannot stand alone since it is not possible to group children based on PA intensity or locate larger groups of children’s activities and locations.

Mixing accelerometer, GPS, participant observation and go-along group interviews created the opportunity to conduct an in-depth exploration of children divided in a Low, Middle and High PA group, which can aid development of interventions targeting specific groups of children in the school environment. Moreover, using the Five W Questions as an analytic tool in the analysis of the data facilitated a coherent and structured mixing process that insured an in-depth exploration.

Even though it limited the generalizability we deliberately chose to pilot test the combination of methods focusing on a single school [76, 77]. The argument was to explore the benefits of using the elaborate combination of methods before encompassing on this effort in a larger study including schools from contrasting areas [47]. Replication of the mixed methods in other western schools would be required to further explore PA behavior during recess.

## Conclusions

This study contributes to the current literature by an in-depth examination of the PA behavior among a Low, Middle and High PA group of children during recess, using a mixed methods approach. We found that combining quantitative and qualitative methods in exploring children’s PA behavior during recess was a valuable approach that did not merely duplicate data but offered complementary insights and understandings that may be difficult to assess through reliance on a single method of data collection. Using a mixed-methods approach to investigate children’s PA behavior during recess helped gain in-depth knowledge that can aid development of future interventions in the school environment.
